# Public awareness of and attitudes towards research biobanks in Latvia

**DOI:** 10.1186/s12910-020-00506-1

**Published:** 2020-07-31

**Authors:** S. Mezinska, J. Kaleja, I. Mileiko, D. Santare, V. Rovite, L. Tzivian

**Affiliations:** 1 Institute of Clinical and Preventive Medicine, University of Latvia, Rainis Boulevard 19, Riga, LV-1586 Latvia; 2 Latvian Biomedical Research and Study Centre, Ratsupites Str. 1-k1, Riga, LV-1067 Latvia

**Keywords:** Research biobanks, Public opinion, Public attitudes, Informed consent, Latvia

## Abstract

**Background:**

Public awareness and engagement are among the main prerequisites for protecting the rights of research participants and for successful and sustainable functioning of research biobanks. The aim of our study was to analyse public awareness and attitudes towards research biobanks in Latvia, and to compare these data with the results of the 2010 Eurobarometer study. We also analysed the influence of awareness and attitudes towards biobanks on willingness to participate in biobank studies and on preferred type of informed consent.

**Methods:**

We developed a 12-question survey repeating seven questions about biobanks from the 2010 Eurobarometer questionnaire and adding five others. After describing the study variables, we performed a two-stage analysis of the results. In the first stage we analysed differences between the answers from 2010 and 2019 and conducted univariate analyses of relationships among particular variables, and between those variables and the socio-demographic characteristics of participants. In the second stage we investigated multivariable associations of willingness to participate and type of consent with awareness, trust and the socio-economic characteristics of participants.

**Results:**

According to our study, the general public in Latvia is still not well informed about research biobanks. Fewer respondents have heard about research biobanks than in 2010. At the same time, the number of respondents who are willing to donate biological samples and personal data to a biobank has increased, e.g. the number of respondents who would definitely or probably be willing to provide information about themselves has increased from 25.8.% to 40.7 since 2010. Overall, concerns about the donation of different types of biological samples and data to a biobank have slightly decreased.

**Conclusions:**

Public awareness about biobanks is important for their sustainability. It needs to be increased not only by traditional methods of informing the public, but also by more innovative and participatory approaches, e.g. by citizen science projects. There is a need to strengthen the public visibility and trustworthiness of ethics committees in Latvia in the field of biobanking.

## Background

Latvia is a European Union (EU) member state with several actively functioning research biobanks. The oldest and one of the most active is the national Genome Database of Latvian Population established in 2003 and currently storing biological samples from more than 35,000 donors (Latvia had 1,919,968 residents in 2019). The national biobank community faces the need to integrate the national network of biobanks in the European Research Area to promote international collaboration in biomedical research. The integration process started when Latvia was approved as a member of the Biobanking and Biomolecular Resources Research Infrastructure (BBMRI-ERIC) in 2016. In 2018, Latvia signed the declaration “Towards access to at least one million sequenced genomes in the European Union by 2022”, which aims to link genomic health data throughout the EU [1]. In general, Latvia has most of the prerequisites for further development of research biobanking: human resources, knowledge capital, infrastructure and national network of biobanks. At the same time, there are significant gaps in existing legal frameworks and state policies that prevent the successful functioning of biobanks, and only one biobank – the Genome Database of Latvian Population - is clearly regulated by the law [2]. Additionally, there are basic national regulations defining the standards for collection, storage, use and transfer of biological samples of human origin for use in biomedical research; however, most of the laws and regulations in force were developed more than 15 years ago and do not meet current international standards. An appropriate solution would be to strengthen the legal framework in Latvia by adopting a new law on biobanks, and a national working group established by the Latvian National Node coordinating BBMRI-ERIC activities has started the process of drafting a new Biobank law. Also, the research ethics committee system still needs improvements regarding transparency and procedural clarity of ethical review, conflicts of interest management, composition of committees and motivation of their members [3].

Public awareness and engagement are among the main prerequisites for protecting the rights of research participants, developing new regulations, and for successful and sustainable functioning of research biobanks [4]. Budin-Ljøsne et al. in their analysis of ethical, legal and social issues in the context of national biobank infrastructures include “lack of knowledge surrounding biobank research among the general public” and “lack of public debate” among the major social/political challenges, and suggest organising public forums and informing society as strategies to address those challenges [5]. The World Medical Association (WMA) Declaration of Taipei emphasises transparency, participation, inclusion and accountability among the principles of biobank governance, fostering the trustworthiness of biobanks [6]. Implementation of these principles cannot be one-sided; it requires all stakeholders, including the general public, to become involved. Therefore, analysis of public opinion by measuring public awareness, attitudes and willingness to participate is very important for giving the public a voice in debates. However, it does not substitute for more participatory methods of public engagement.

In 2010, a pan-European Eurobarometer survey of Life Sciences and Biotechnology included a set of questions on public attitudes towards biobanks [7]. The survey was conducted in February 2010 and used multi-stage probability sampling to obtain representative samples in each EU member state as well as Croatia, Iceland, Norway, Switzerland and Turkey [8] (ca.1000 respondents in each country; 1013 in Latvia). The questions on biobanks “were administered to half of the sample in each country through a randomised split ballot” [8]. The findings of this study showed that “the publics of Europe are heterogeneous in their response to biobanks” [8]; people in Eastern and Southern Europe were less willing to participate in research biobanks and preferred narrow (specific) consent [8]. The low willingness to participate in biobanks in Latvia as shown by this 2010 Eurobarometer study may be at least partially explained by the general level of trust in science in the country which in 2010 was lower than average in the EU [9].

These 2010 findings, and a small qualitative interview study on donors’ attitudes towards the Genome Database of Latvian Population [10], have been the only data on public attitudes towards biobanks in Latvia so far. To fill this knowledge gap in the context of developing the new Biobank law, we decided to analyse public attitudes by conducting a survey repeating the 2010 Eurobarometer survey questions on biobanks [7]. We added questions on awareness of the Genome Database of Latvian Population, willingness to donate specific types of biological samples, and preferred types of consent for donation of surplus surgical material, to the 2010 questions. The aim of our study was to analyse public awareness and attitudes towards research biobanks in Latvia, and to compare these data with the results of the 2010 Eurobarometer study. We also analysed the influence of awareness and attitudes towards biobanks on willingness to participate in biobank studies and preferred type of informed consent on the basis of the new 2019 survey data.

## Methods

A 12-question quantitative survey was developed by the panel of six authors. The survey included all seven questions on biobanks and the introductory explanation of the term ‘biobank’ from the 2010 Eurobarometer questionnaire (question numbers B12-B18 in that original questionnaire), which together with its translations and data is available in open access on the GESIS archive [11]. The 2010 translations of the Eurobarometer questionnaire into Latvian and Russian were used for the 2019 survey. Five additional questions on willingness to donate specific types of biological samples (blood, surplus surgical material, urine, faeces), opt-out consent for biobanking surplus surgical material, and two questions on awareness about the Genome Database of Latvian Population were added to the 2019 survey by the research group.

Our 2019 survey was conducted in March 2019 and used multi-stage stratified random sampling to obtain a representative sample of the general population of Latvia. Data were collected by the research centre SKDS as part of the monthly Omnibus survey by conducting face-to-face interviews in the respondents’ homes. The sample comprised Latvian residents aged 18 to 75 years. Sample design was based on the latest statistics on permanent residents of Latvia.

### Awareness and willingness to participate

Awareness of biobanks in the 2010 Eurobarometer survey was assessed by answers to three questions: (1) *Before today, have you ever heard anything about biobanks?* (2) *Have you ever talked about biobanks with anyone before today?* (3) *Have you ever searched for information about biobanks?* These questions were then combined to create the next levels of categories: (a) passive engagement (heard but not talked or searched for information), (b) active engagement (heard and talked or searched for information), and (c) not heard. In addition to those three questions, we included two new questions concerning the Genome Database of Latvian Population in the 2019 survey: (1) *Have you ever heard anything about the Genome Database of Latvian Population?* with answers ‘yes’, ‘no’ and ‘difficult to say’; (2) *How would you rate the work of the Genome Database of Latvian Population?*, where the possible answers were ‘in favour of the idea’, ‘wait-and-see attitude’, ‘need more information’, ‘never heard’, ‘against it’, and ‘cannot comment’.

Willingness to participate in biobank studies was measured by the 2010 Eurobarometer survey question: *Would you be willing to provide information about yourself to a biobank?* and the possible answers were: ‘yes, definitely’, ‘yes, probably’, ‘no, probably not’, ‘no, definitely not’. These answers were combined for analysis into two more general ‘yes’ and ‘no’ categories. In addition to this question from the 2010 questionnaire, we added a question about participants’ willingness to donate specific types of biological material to a biobank in 2019: *Would you agree to including the following samples of your biological material into a biobank?* mentioning ‘blood’, ‘surplus surgical material’, ‘urine’ and ‘faeces’ as types of biological material and providing ‘yes’, ‘no’, ‘do not know’ as possible answers for each type. As each participant could choose multiple answers for this question, we descriptively analysed the answers about each type of biological samples individually.

### Informed consent

To explore the opinions of respondents about the type of informed consent we used two Eurobarometer 2010 questions: (1) *When a scientist does research on data in a biobank, what do you think about the need for this kind of permission?* (there was an explanation of the term ‘informed consent’ before this question), and the possible answers were: ‘no need to ask for permission’, ‘ask for permission only once’, ‘ask for permission for every new piece of research’. For statistical analysis, this question was re-coded into two categories: (a) broad consent (‘no need to ask permission’ or ‘ask only once’) and (b) narrow consent (‘ask for permission for every new piece of research’). The second question was: (2) *Would you agree that your surplus surgical material would be included in a biobank without your consent, if you would not specifically prohibit it?* suggesting four options of answers: ‘yes, definitely’, ‘yes, probably’, ‘no, probably not’, ‘no, definitely not’. Answers on this question were dichotomised for analysis into two types: (a) yes/opt-out and (b) no/opt-in.

### Concerns

To investigate participants’ concerns about the collection of their data and samples for a research biobank, we used answers to a Eurobarometer 2010 question: *Would you personally be concerned or reluctant about the collection of any of the following types of data and materials from you?* As the participants could choose multiple answers to this question, we performed descriptive statistics for each of the yes/no answers for blood samples; tissue collected during medical operations; genetic profile; medical records; and lifestyle.

### Governance and trust

To investigate participants’ trust and attitudes towards governance of research biobanks we used two Eurobarometer 2010 questions: (1) *Who do you think should be primarily responsible for protecting the public interest?* and (2) *Do you think the sharing and exchange of personal data and biological materials across Member States should be encouraged?* For the question on responsibility, descriptive statistics for primary and secondary responsibility was performed. For the question on sharing samples across EU, we dichotomised the answers (‘yes, definitely’, ‘yes, probably’, ‘no, probably not’, ‘no, definitely not’) into yes/no groups.

### Statistical analysis

Descriptive statistics was performed for all study variables. Variables, numbers and percentages were presented for categorical variables. For qualitative variables, mean and standard deviation were presented if the variable was normally distributed, and median and interquartile range otherwise.

After description of study variables, we analysed our results in two stages. In the first stage, we analysed differences between the answers from 2010 and 2019 for the following 2019 survey answers: awareness of research biobanks, willingness to participate, opinions regarding the type of informed consent, concerns regarding donation of samples and data, and governance and trust. We performed univariate analyses of the relationships among these variables by themselves, and between these variables and the socio-demographic characteristics of participants.

In the second stage, we investigated multivariable associations of willingness to participate and type of consent with awareness, trust and the socio-economic parameters of participants for the 2019 data. As in the 2010 Eurobarometer survey [8], for the 2019 survey we built multivariable logistic regression models to determine the role of awareness and trust in predicting the odds of being willing to donate samples and data to a biobank and preferring broad consent over narrow consent. To measure awareness in these models, we combined the answers to the questions on awareness and searching for information - ‘passive engagement’, ‘active engagement’, and ‘not heard’. For trust, we used the question on sharing the information across EU Member States (dichotomous variable, yes/no). All models were adjusted for participant age, years of education, and gender. SPSS software, v. 26, was used for statistical analyses. *P* values < 0.05 were considered statistically significant.

## Results

### Demographic characteristics of the respondents

One thousand and seventeen respondents participated in our survey (*N* = 1017). Their mean age was 46.3 years (standard deviation, SD = 15.8). Slightly more women than men participated, and most of the participants were married, with secondary or professional education, and were native Latvians. Most earned less than 210 Euro per month per person in the family (median 301–400 Euro), had no children, worked in the private sector and lived in cities outside the capital (Table 1). There were no significant differences between the participants in the Eurobarometer 2010 and our 2019 surveys in respect of gender or marital status, but the 2019 participants were significantly older, with fewer living in rural areas, and with more Russian speakers (Supplement Table 1).

**Table 1:** Socio-demographic characteristics of respondents

Variable	Category	2019 results
Gender (N, %)	Male	480 (47.2)
	Female	537 (52.8)
Age, median (mean ± SD)		46.0 (46.3 ± 15.8)
Marital status (N, %)	Single	196 (19.3)
	Married	600 (59.0)
	Divorced	131 (12.9)
	Widowed	90 (8.8)
Education (N, %)	Primary	116 (11.4)
	Secondary/ professional	631 (62.0)
	Higher	270 (26.5)
Average salary per month per person in the family (Euro)	< 210	203 (22.8)
	211–300	180 (20.2)
	301–400	190 (21.4)
	401–590	137 (15.5)
	> 591	179 (20.1)
Having children under the age of 18 (N, %)		348 (34.2)
Nationality (N, %)	Latvian	601 (59.1)
	Russian	327 (32.2)
	Other	89 (8.8)
Residential status (N, %)	Latvian citizen	873 (85.8)
	Latvian non-citizen^a^	144 (14.2)
Working status (N, %)	Governmental sector	195 (19.2)
	Private sector	455 (44.7)
	Not working	367 (36.1)
Place of residence (N, %)	Capital city	335 (32.9)
	Another city	385 (37.9)
	Rural area	297 (29.2)

^a^‘Non-citizens’ is a special legal status established in 1991 for former USSR citizens permanently residing in Latvia without the citizenship of the Republic of Latvia or any other country

### Awareness and willingness to participate

In 2019, 262 (25.8%) of the 1017 participants in the study said they had heard about biobanks. Among those, 100 (38.2%) were actively engaged in searching information on biobanks, but the others (*N* = 162) were not. One hundred and ninety-six participants (19.2%) said they have heard about the Genome Database of Latvian Population. Most of them stated that they ‘need more information’ to rate the work of the Genome Database of Latvian Population (*N* = 61, 6.0%), or are ‘in favour of the idea” (*N* = 58, 5.7%) or have a ‘wait-and-see attitude’ (*N* = 53, 5.2%). Only one person was definitely against this project (0.1%), and a further 23 (2.3%) had no opinion or did not answer this question.

Awareness of biobanks in the 2019 survey was significantly related to the education and income of participants, their residential status and place of work. Participants who lacked awareness were of lower educational level, and only one participant with primary education was actively engaged in searching information about biobanks. There was no difference in active and passive engagement between those whose average salary per month per family member was over 590 Euro, but they were more engaged, both passively and actively, than participants with lower salaries. Those participants with lower salaries were more likely to be actively than passively engaged. More Latvian citizens than Latvian non-citizens were aware of biobanks, and more Latvian citizens were actively engaged (Supplement Table 2).

Among all participants in the 2019 survey, 373 (36.7%) would definitely (*N* = 67; 6.6%) or probably (*N* = 306; 30.1%) be willing to provide information about themselves to a biobank, while 544 (53.5%) would not be willing to do so (of those, *N* = 250 (24.5%) were definitely not willing to provide information). One hundred participants (9.8%) did not answer this question. We observed differences in willingness to participate with all socio-demographic variables excluding gender, education, and having children under the age of 18. Participants who were willing to participate in biobank research by sharing their information with a biobank were younger, most of them earned more than 590 Euro per month per one family member, and more were native Latvians with Latvian citizenship (Supplement Table 3).

Among all 2019 survey participants, 462 (45.4%) were willing to donate blood samples to a biobank; 409 (40.2%) to provide surplus surgical material; 430 (42.2%) to donate urine samples; and 411 (40.4%) to donate faeces samples.

### Informed consent

Broad consent for donation of biological samples to a biobank was preferred by only 27.4% of all participants; 62.2% preferred narrow consent; 105 (10.3%) had no opinion on this question. Opinions regarding the type of informed consent were related to participants’ education (more people with higher education chose broad consent), income (more people with higher income preferred broad consent) and place of residence (Supplement Table 4).

An opt-out approach for surplus surgical material was approved by 39.3% of participants, while more respondents would prefer an opt-in form of informed consent for samples of this type (53.1%). Opt-out use of surplus surgical material was related to education (more people with higher education would allow such use), residential status, working status, and place of residence (most respondents from cities other than the capital preferred not to allow use) (Supplement Table 5).

Most of the participants (59.6%) agreed to sharing of personal data and biological samples across EU Member States (21.2% were definitely sure about it); 19.0% found it difficult to answer this question. A positive attitude towards sharing of biobank samples among EU states was related to marital status, average salary, residential status, and place of living (Supplement Table 6).

### Biobank-related opinions and socio-economic status of survey participants

All investigated biobank-related parameters were univariately related to average monthly salary and place of residence. Participants with higher salaries were more aware of biobanks, more willing to participate, less open to using broad consent, but more willing to share data and samples among EU member states. Those living in small cities were more aware of biobanks, less willing to participate, preferred narrow consent, were less in favour of opt-out use of surplus surgical material, and were less willing than the other two groups to share data and samples among EU member states (Supplement Tables 1–6).

Additional differences were observed among participants with different educational levels and working status. More educated participants were more aware of biobanks (both passively and actively), and more agreed to opt-out use of surplus surgical material (Supplement Tables 2 and 5). There were no differences among participants in respect of level of education in their willingness to participate, type of preferred consent (broad versus narrow) or willingness to share data and samples among EU member states (Supplement Tables 3, 4 and 6). Those participants who worked were more aware of biobanks, more willing to participate, and more willing to accept the opt-out use of surplus surgical material (Supplement Tables 2, 3, 5). There were no differences related to working status in the type of preferred consent or willingness to share data and samples among EU member states (Supplement Tables 4 and 6).

There was a relationship between awareness of biobanks and engagement and willingness to participate (*p* <  0.01). Those who were actively engaged were more willing to participate. We also observed a relationship between willingness to participate and the preferred form of consent (*p* <  0.01) (Table 2).

**Table 2:** Relationship between willingness to participate, awareness, engagement, and the preferred form of consent

		Willingness to participate		***P*** value
		Yes	No	
**Awareness**	Passively engaged, *N* = 153	76 (49.7)	77 (50.3)	< 0.01
	Actively engaged, *N* = 95	64 (67.4)	31 (32.6)	
	Not heard, *N* = 669	232 (34.7)	437 (65.3)	
**Type of consent**	Broad, *N* = 259	157 (60.6)	102 (39.4)	< 0.01
	Narrow, *N* = 575	203 (35.3)	372 (64.7)	

### Concerns

Most participants had no concerns regarding donation of their biological samples and personal data to a biobank. This applied to all types of biological material mentioned in the question, and to personal data (Table 3).

**Table 3:** Concerns about donation of biological samples and data to biobanks

Materials for biobanks	Concerns, n = 1017	
	No (N, %)	Yes (N, %)
Blood samples	758 (74.5)	259 (25.5)
Tissue collected during medical operations	745 (73.3)	272 (26.7)
Genetic profile	738 (72.5)	279 (27.5)
Medical records	655 (64.4)	362 (35.6)
Lifestyle information	755 (74.2)	262 (25.8)
Other	968 (95.1)	49 (4.9)

### Governance and trust

When they answered the question about stakeholders responsible for protecting the public interest in the context of biobank research, most participants agreed that the first responsible group are medical doctors, and the second are researchers. For almost 16% of the participants it was difficult to decide about the first and second responsible stakeholder (Table 4).

**Table 4:** Stakeholders responsible for protecting public interests

Responsible stakeholder	First responsibility (N, %)	Second responsibility (N, %)
Medical doctors	293 (28.8)	144 (14.1)
Researchers	158 (15.6)	219 (21.6)
Public institutions (universities, hospitals)	36 (3.6)	73 (7.2)
National governments	130 (12.7)	97 (9.6)
Ethics committees	26 (2.5)	45 (4.4)
International organisations such as the EU or WHO	76 (7.5)	80 (7.9)
National Data Protection Authorities	155 (15.2)	172 (16.9)
Difficult to say	121 (11.9)	39 (3.8)

### Differences between results of 2010 and 2019 surveys

There were statistically significant differences between results of the 2010 and 2019 surveys regarding awareness of biobanks, willingness to participate, type of consent, and sharing data and samples among EU member states (Table 5). In 2019, more participants had not heard about biobanks, but among those who had heard about biobanks in 2010 and 2019 there were no differences in passive and active engagement (*p* = 0.39). In 2019, more participants were willing to participate, preferred broad consent, and were positive about sharing data and samples among EU member states (Table 5).

**Table 5:** Differences between 2010 and 2019 survey results.

Variable	Category	2010 survey	2019 survey	***P*** value
Awareness, N (%)	Passively engaged	134 (27.6)	162 (15.9)	< 0.01
	Actively engaged	87 (17.9)	98 (9.6)	
	Not heard	265 (54.5)	757 (74.4)	
Willingness to participate, N (%)	Yes	117 (25.8)	373 (40.7)	< 0.01
	No	337 (74.2)	544 (59.3)	
Type of consent, N (%)	Broad	88 (20.2)	279 (30.6)	< 0.01
	Narrow	347 (79.8)	632 (69.4)	
Sharing of data and samples within EU, N (%)	Yes	248 (59.8)	606 (73.5)	< 0.01
	No	167 (40.2)	218 (26.5)	

### Multivariate analysis

**Fig. 1:**
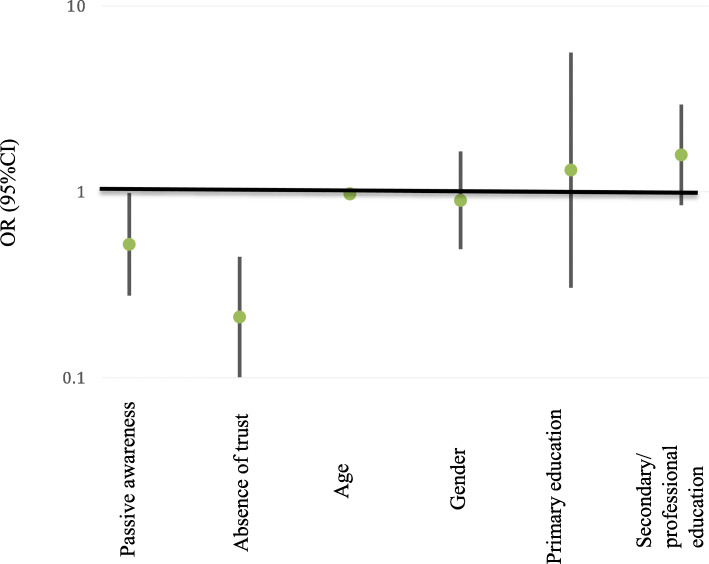
Association of willingness to participate in biobank with awareness, trust and socio-demographic factors – results of logistic regression model (ORs are presented at logarithmic scale)

In fully adjusted multivariable regression models of the 2019 survey results, willingness to participate was significantly associated with awareness, trust and age (adjusted R^2^ = 17.8) (Fig. 1, Supplement Table 7). Passive awareness (odds ratio, OR = 0.52 [95% confidence interval, CI 0.28, 0.96]), absence of trust (OR = 0.21 [CI 0.10; 0.44]), and older age (OR = 0.97 [CI 0.95; 0.99]) reduced willingness to participate. No other investigated factors were associated with willingness to participate.

**Fig. 2:**
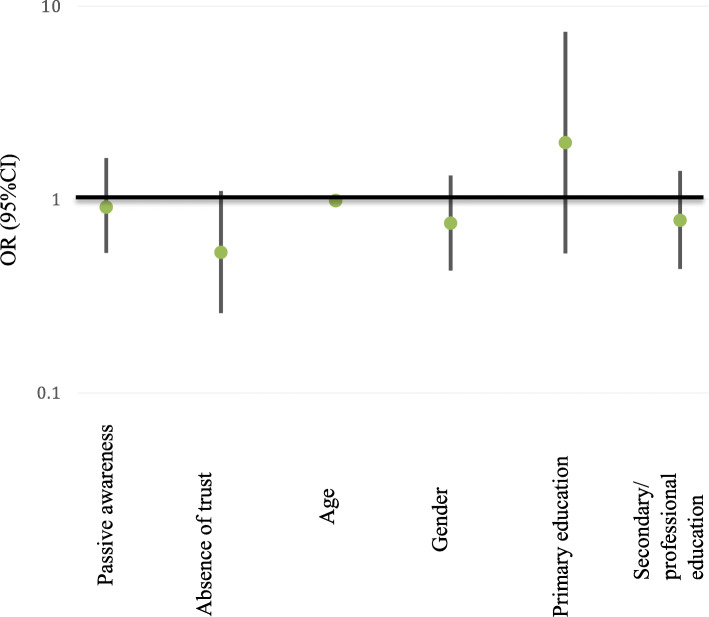
Association of preferred type of informed consent (broad over narrow) with awareness, trust and socio-demographic factors – results of logistic regression model (ORs are presented at logarithmic scale)

We found no significant association between preferred type of informed consent and any socio-demographic factor (Fig. 2, Supplement Table 8). However, the overall tendency remains as in the previous regression model and all factors except secondary/professional education were in the same direction. Absence of trust reduced non-significantly a probability for broad consent (OR = 0.54 [0.27; 1.08]). Age did not affect this probability but remains in the same direction as in the previous model (OR = 0.99 [0.97; 1.01]).

## Discussion

According to our study, the general public in Latvia is still not well informed about research biobanks. The number of respondents who have heard about research biobanks is less than in 2010. However, more respondents are willing to donate biological samples and personal data to a biobank, e.g. the number of respondents who would definitely or probably be willing to provide information about themselves to a biobank has increased from 25.8.% to 40.7 since 2010. Overall, concerns about the donation of different types of biological samples and data to a biobank have slightly decreased, but the greatest concern remains the use of data from patients’ medical records.

A recent literature review [4] and studies in other European countries also show willingness to donate samples and information to biobanks despite low awareness of them. In a regional study conducted in Germany in 2015 the respondents showed high overall support for biobanks, e.g., “70.4% of respondents would be willing to donate biomaterial to a biobank during a hypothetical stay in hospital”; however, only about one third (30.8%) had previously heard of biobanks, and this level of awareness had not changed since the 2010 Eurobarometer study [12].

At the same time, rising awareness is not a panacea for promoting willingness to donate samples and personal data. The relationship between high levels of awareness, positive attitudes and real participation rates in biobanks is not direct. As emphasised by Snell and Tarkkala in their analysis of the rhetoric of a ‘willing population’ in Finnish biobanking: “supporting and positive attitudes or high levels of general trust in a given society are not in themselves straightforward indications of people’s actual willingness and preparedness to participate in biobanking” [13]. Such criticisms highlight the need to seek new ways of promoting the values of transparency, participation, inclusion and accountability and of overcoming the risk of instrumentalisation of donors by looking at potential donors as just a resource providing samples and personal data to a biobank or passive objects of research requiring protection. One possible solution suggested by Langhof et al. is that biobanks should “concentrate on how to balance the different interests of patients/donors, (public) funding agencies, clinician/researcher collecting, and biobank staff processing and storing human biological materials and, thus, acting as stewards of the hosted biosamples” [14]. However, this approach, which involves stakeholders but still nominates scientists as the main responsible stewards of samples and data, could be outdated in the context of biobanking. As shown in other fields of science, e.g. management of open data [15] or environmental science [16, 17], broader understanding of stewardship moving towards active engagement of stakeholders can be highly beneficial, allowing researchers to focus on the needs of the public and communities and to avoid instrumentalising attitudes. Another approach suggested by Gottweis et al. is “to create some sort of expert publics, i.e., publics composed of people who are well informed about a certain issue at stake” [18]. This approach might be combined with previously mentioned broader understanding of stewardship to build new forms of collaborative biobank governance.

The ongoing process of development of the new Biobank law in Latvia includes public consultations with stakeholders (general public, scientists, industry, non-governmental organizations etc); however involvement of and collaboration with stakeholders can be made even more active by implementing community-based participatory research methods and citizen science approaches for choosing research priorities and involving donors more actively in the biobanking process. Recent studies demonstrate various participatory approaches, e.g. methods for improving informed consent [19], wiki-governance models [20], citizen science in the form of personal genome projects [21], and many other methods, opening a promising new perspective on biobanking. Some recent examples of biobank-based participatory research show “enthusiastic response for ‘taking part’ and ‘being listened to’” [22] and raise the hope that “citizen science applications and participatory research and governance strategies could lead into a novel area to explore for the field” [23]. As emphasised by Tupasela et al., by using participatory models, “increased participation also increases the flow of information in both directions, as opposed to being top-down in nature” [24]. Although some critical views indicate that most public members have “neither the time nor the interest to become involved in participatory structures of biobank designs” [18] and genuine participation seems more rhetoric than reality [25], the existing examples are promising, especially if the central participatory elements (being educational, promoting a sense of being involved and degree of control [25]) are respected.

The preferred and ethically most justifiable type of informed consent remains one of the most discussed ethical issues in the context of biobanking. The ‘communitarian turn’ in bioethics, and the ethics of biobanking in particular [26, 27], emphasised the importance of broad consent and solidarity for maximising public benefit in the context of biobanking. In Latvia, biobanks currently use broad consent for a wide range of unspecified future research including an option to restrict particular types of sample use. However, the results of our survey show that most respondents still prefer narrow consent, providing information about each particular research study where the donor’s samples are used. A possible solution could be to introduce dynamic consent, “a digital decision-support where modern IT communication strategies are used to continuously inform and offer choices to donors to specify the types of research for which their specimens can be used or not” [4]. However implementation of this type of consent in Latvia is limited by the financial recourses of biobanks. Some authors also warn about risks that use of dynamic consent based on digital technologies could lead to deepening the ‘digital divide’ by favouring those “with knowledge and access to digital technologies” [28], which in turn could lead to negative effects and decrease participant engagement in research. Therefore, it is important to look for solutions likely to improve participant inclusivity and to evaluate empirically how dynamic consent tools will affect equality in access to research participation [28].

We should admit that our study has some limitations related to the sample. Compared to the 2010 Eurobarometer study sample there are slight differences in the respondents’ ages, which could affect comparisons of the results since younger people are likely to be more aware of biobanks. Similarly, slightly more residents of rural areas were included in the 2019 sample, and this could also have affected the results of our study.

## Conclusions

In comparison with 2010, there is less awareness of research biobanks among the general public in Latvia, but the public is slightly more willing to participate in research biobanks by donating biological samples and personal data. Younger people with higher educational levels who are more engaged in searching information about biobanks are more willing to donate samples. This shows a need to inform a broader public including the older generation and people in rural areas about the role of research biobanks. Public awareness about biobanks is important for their sustainability and it needs to be increased not only by traditional methods of informing the public, but also by more innovative and participatory approaches, e.g. by citizen science projects. Biobanks should not only promote donors’ awareness, but also increase willingness to donate material and to engage in biobank-based research by active participation. More information on biobanks and biobank-based research should be made available to the public in Latvia to demonstrate the results and benefits of donations.

Another important issue is responsibility for protecting the public interest. Most respondents in our survey chose physicians and scientists as responsible actors for protecting public interest in the field of biobanks. Researchers and physicians were also the groups most trusted across Europe in the 2010 Eurobarometer survey [7]. The main responsibility in our 2019 survey was attributed to individual doctors and scientists, not to the institutions for which they work or other institutions such as ethics committees or governmental bodies. The attribution of responsibility to scientists has reduced since 2010, but the attribution of responsibility to national data protection authorities has significantly increased, which can most likely be explained by the introduction of General Data Protection Regulation and the public discussions surrounding this process. These results show a lack of information about or trust in research ethics committees in Latvia, which needs clarification by further research and a discussion about possible ways of strengthening the role of ethics committees in the context of development of the new Biobank law. Currently, the public in Latvia sees doctors and scientists as the main responsible and trustworthy actors for protecting public interest, but the public either does not trust research ethics committees or is not aware on their role in protecting public interests. There is a need to strengthen the public visibility and trustworthiness of ethics committees in Latvia in the field of biobanking.

## Supplementary information

**Additional file 1: Supplement Table 1.** Socio-demographic differences between participants of 2010 and 2019 surveys.

**Additional file 2: Supplement Table 2.** Relationships between awareness on biobanks and socio-demographic characteristics of participants of 2019 survey.

**Additional file 3: Supplement Table 3.** Relationships between willingness to participate and socio-demographic characteristics of participants of 2019 survey.

**Additional file 4: Supplement Table 4.** Relationships between the opinion regarding the type of informed consent and socio-demographic characteristics of participants of 2019 survey.

**Additional file 5: Supplement Table 5** Relationships between opt-out use of surplus surgical material and socio-demographic characteristics of participants of 2019 survey.

**Additional file 6: Supplement Table 6.** Relationships between attitude towards sharing of biobank samples among EU states and socio-demographic characteristics of participants of 2019 survey.

**Additional file 7: Supplement Table 7.** Association of willingness to participate in biobank with awareness, trust and socio-demographic factors – results of logistic regression model.

**Additional file 8: Supplement Table 8.** Association of preferred type of informed consent (broad over narrow) with awareness, trust and socio-demographic factors – results of logistic regression model.

## Data Availability

The datasets generated and analysed during the current study are available in the OSF repository, DOI: 10.17605/OSF.IO/B34R5 2010 Eurobarometer data are available in open access in the GESIS archive (registration is required) 10.4232/1.11428.

## Abbreviations

BBMRI-ERIC: Biobanking and Biomolecular Resources Research Infrastructure
CI: confidence interval
EU: European Union
OR: odds ratio
SD: standard deviation
WMA: World Medical Association
