# Supplementary material for: Public awareness of and attitudes towards research biobanks in Latvia
Source: BMC Med Ethics. 2020 Jul 31;21:65. doi: 10.1186/s12910-020-00506-1 (PMC7393882; doi:10.1186/s12910-020-00506-1)
Supplement: Supplementary file 1 — Additional file 1: Supplement Table 1. Socio-demographic differences between participants of 2010 and 2019 surveys. [file 12910_2020_506_MOESM1_ESM.docx]

Supplement Table 1. Socio-demographic differences between participants of 2010 and 2019 surveys

| **Variable** | **Category** | **2010 survey** | **2019 survey** | ***P* value** |
| --- | --- | --- | --- | --- |
| Gender, N (%) | Male  Female | 468 (46.2)  545 (53.8) | 489 (48.1)  528 (51.9) | 0.34 |
| Age, median (mean ± SD) |  | 42.0  (42.4 ± 17,2) | 46.0  (46.3 ± 15.8) | < 0.01 |
| Marital status, N (%) | Single  Married  Divorced  Widowed | 195 (21.2)  569 (61.8)  92 (10.0)  65 (7.1) | 196 (19.3)  600 (59.0)  131 (12.9)  90 (8.8) | 0.07 |
| Place of residence, N (%) | Capital city  Another city  Rural area | 343 (33.9)  303 (30.0)  365 (36.1) | 335 (32.9)  385 (37.9)  297 (29.2) | < 0.01 |
| Main language | Latvian  Russian  Other | 796 (78.6)  217 (21.4)   1. (0.0) | 618 (60.8)  394 (38.7)  5 (0.5) | < 0.01 |
