# Supplementary material for: Public awareness of and attitudes towards research biobanks in Latvia
Source: BMC Med Ethics. 2020 Jul 31;21:65. doi: 10.1186/s12910-020-00506-1 (PMC7393882; doi:10.1186/s12910-020-00506-1)
Supplement: Supplementary file 2 — Additional file 2: Supplement Table 2. Relationships between awareness on biobanks and socio-demographic characteristics of participants of 2019 survey. [file 12910_2020_506_MOESM2_ESM.docx]

Supplement Table 2. Relationships between awareness on biobanks and socio-demographic characteristics of participants of 2019 survey

|  |  | **Awareness** | | |  |
| --- | --- | --- | --- | --- | --- |
| **Variable** | **Category** | **Passively engaged,**  **N = 162** | **Actively engaged,**  **N = 100** | **Not heard,**  **N = 757** | ***P* value** |
| Gender (N, %) | Male  Female | 67 (13.7)  94 (17.8) | 52 (10.7)  47 (8.9) | 369 (75.6)  387 (73.3) | 0.16 |
| Age,  Mean (SD) |  | 46.7 (14.7) | 42.9 (13.4) | 46.3 (16.1) | 0.10 |
| Marital status (N, %) | Single  Married  Divorced  Widowed | 26 (13.4)  103 (17.0)  22 (16.7)  11 (12.5) | 24 (12.4)  66 (10.9)  7 (5.3)  3 (3.4) | 144 (74.2)  436 (72.1)  103 (78.0)  74 (84.1) | 0.06 |
| Education (N, %) | Primary  Secondary/ professional  Higher | 11 (9.7)  96 (15.2)  54 (19.7) | 1 (0.9)  50 (7.9)  48 (17.5) | 101 (89.4)  484 (76.8)  172 (62.8) | < 0.01 |
| Average salary per month per person in the family (Euro) | < 210  211 – 300  301 – 400  401 – 590  > 591 | 29 (14.4)  29 (16.2)  29 (15.3)  22 (16.1)  41 (22.9) | 11 (5.4)  6 (3.4)  17 (8.9)  13 (9.5)  39 (21.8) | 162 (80.2)  144 (80.4)  144 (75.8)  102 (74.5)  99 (55.3) | < 0.01 |
| Having children under the age of 18 (N, %) | Yes  No | 60 (17.0)  102 (15.4) | 44 (12.5)  55 (8.3) | 248 (70.5)  507 (76.4) | 0.17 |
| Nationality (N, %) | Latvian  Russian  Other | 105 (17.5)  46 (14.0)  11 (12.4) | 63 (10.5)  24 (7.3)  11 (12.4) | 431 (72.0)  259 (78.7)  67 (75.3) | 0.15 |
| Residential status (N, %) | Latvian citizen  Latvian non-citizen | 148 (17.0)  14 (9.7) | 93 (10.7)  6 (4.2) | 632 (72.4)  124 (86.1) | < 0.01 |
| Working status (N, %) | Governmental sector  Private sector  Not working | 35 (17.9)  78 (16.8)  49 (13.6) | 29 (14.9)  48 (10.4)  22 (6.1) | 131 (67.2)  337 (72.8)  288 (80.2) | < 0.01 |
| Place of residence (N, %) | Capital city  Another city  Rural area | 44 (13.0)  64 (16.7)  54 (18.4) | 51 (15.0)  20 (5.2)  27 (9.2) | 244 (72.0)  300 (78.1)  212 (72.4) | < 0.01 |
