# Supplementary material for: Public awareness of and attitudes towards research biobanks in Latvia
Source: BMC Med Ethics. 2020 Jul 31;21:65. doi: 10.1186/s12910-020-00506-1 (PMC7393882; doi:10.1186/s12910-020-00506-1)
Supplement: Supplementary file 3 — Additional file 3: Supplement Table 3. Relationships between willingness to participate and socio-demographic characteristics of participants of 2019 survey. [file 12910_2020_506_MOESM3_ESM.docx]

Supplement Table 3. Relationships between willingness to participate and socio-demographic characteristics of participants of 2019 survey

|  |  | **Willingness to share information** | |  |
| --- | --- | --- | --- | --- |
| **Variable** | **Category** | **Yes,**  **N = 373** | **No,**  **N = 544** | ***P* value** |
| Gender (N, %) | Male  Female | 176 (39.7)  197 (41.6) | 267 (60.3)  277 (58.4) | 0.31 |
| Age,  Mean (SD) |  | 43.3 (15.4) | 48.2 (15.6) | < 0.01 |
| Marital status (N, %) | Single  Married  Divorced  Widowed | 88 (48.9)  217 (40.3)  42 (35.3)  26 (32.5) | 92 (51.1)  321 (59.7)  77 (64.7)  54 (67.5) | 0.03 |
| Education (N, %) | Primary  Secondary/ professional  Higher | 36 (35.0)  228 (39.8)  109 (45.2) | 67 (65.0)  345 (60.2)  132 (54.8) | 0.16 |
| Average salary per month per person in the family (Euro) | < 210  211 – 300  301 – 400  401 – 590  > 591 | 60 (32.4)  53 (31.9)  70 (40.0)  61 (49.6)  86 (55.1) | 125 (67.6)  113 (68.1)  105 (60.0)  62 (50.4)  70 (44.9) | < 0.01 |
| Having children under the age of 18 (N, %) | Yes  No | 133 (42.2)  182 (57.8) | 240 (40.0)  360 (60.0) | 0.41 |
| Nationality (N, %) | Latvian  Russian  Other | 251 (46.2)  98 (33.2)  23 (29.5) | 292 (53.8)  197 (66.8)  55 (70.5) | < 0.01 |
| Residential status (N, %) | Latvian citizen  Latvian  non-citizen | 342 (43.3)  31 (24.4) | 448 (56.7)  96 (75.6) | < 0.01 |
| Working status (N, %) | Governmental sector  Private sector  Not working | 76 (42.5)  188 (45.7)  109 (33.3) | 103 (57.5)  223 (54.3)  218 (66.7) | < 0.01 |
| Place of residence (N, %) | Capital city  Another city  Rural area | 125 (41.0)  120 (34.0)  128 (49.4) | 180 (59.0)  233 (66.0)  131 (50.6) | < 0.01 |
