# Supplementary material for: Public awareness of and attitudes towards research biobanks in Latvia
Source: BMC Med Ethics. 2020 Jul 31;21:65. doi: 10.1186/s12910-020-00506-1 (PMC7393882; doi:10.1186/s12910-020-00506-1)
Supplement: Supplementary file 4 — Additional file 4: Supplement Table 4. Relationships between the opinion regarding the type of informed consent and socio-demographic characteristics of participants of 2019 survey. [file 12910_2020_506_MOESM4_ESM.docx]

Supplement Table 4. Relationships between the opinion regarding the type of informed consent and socio-demographic characteristics of participants of 2019 survey

|  |  | **Type of consent** | |  |
| --- | --- | --- | --- | --- |
| **Variable** | **Category** | **Broad,**  **N = 279** | **Narrow,**  **N = 633** | ***P* value** |
| Gender (N, %) | Male  Female | 133 (30.6)  146 (30.6) | 302 (69.4)  331 (69.4) | 0.53 |
| Age,  Mean (SD) |  | 45.6 (15.7) | 45.9 (15.3) | 0.72 |
| Marital status (N, %) | Single  Married  Divorced  Widowed | 58 (35.8)  167 (30.0)  33 (28.4)  21 (27.6) | 104 (64.2)  390 (70.0)  83 (71.6)  55 (72.4) | 0.44 |
| Education (N, %) | Primary  Secondary/prof  Higher | 22 (22.9)  166 (29.8)  91 (35.3) | 74 (77.1)  391 (70.2)  167 (64.7) | 0.06 |
| Average salary per month per person in the family (Euro) | < 210  211 – 300  301 – 400  401 – 590  > 591 | 40 (21.7)  51 (32.3)  43 (25.0)  48 (38.1)  65 (38.7) | 144 (78.3)  107 (67.7)  129 (75.0)  78 (61.9)  103 (61.3) | < 0.01 |
| Having children under the age of 18 (N, %) | Yes  No | 103 (31.7)  176 (30.0) | 222 (68.3)  410 (70.0) | 0.70 |
| Nationality (N, %) | Latvian  Russian  Other | 173 (31.5)  83 (28.8)  23 (30.7) | 376 (68.5)  205 (71.2)  52 (69.3) | 0.72 |
| Residential status (N, %) | Latvian citizen  Latvian  non-citizen | 245 (31.1)  34 (27.9) | 544 (68.9)  88 (72.1) | 0.28 |
| Working status (N, %) | Governmental sector  Private sector  Not working | 56 (31.6)  134 (31.3)  89 (29.0) | 121 (68.4)  294 (68.7)  218 (71.0) | 0.75 |
| Place of residence (N, %) | Capital city  Another city  Rural area | 108 (34.7)  84 (25.1)  86 (32.5) | 203 (65.3)  251 (74.9)  179 (67.5) | 0.02 |
