# Supplementary material for: Public awareness of and attitudes towards research biobanks in Latvia
Source: BMC Med Ethics. 2020 Jul 31;21:65. doi: 10.1186/s12910-020-00506-1 (PMC7393882; doi:10.1186/s12910-020-00506-1)
Supplement: Supplementary file 5 — Additional file 5: Supplement Table 5 Relationships between opt-out use of surplus surgical material and socio-demographic characteristics of participants of 2019 survey. [file 12910_2020_506_MOESM5_ESM.docx]

Supplement Table 5. Relationships between opt-out use of surplus surgical material and socio-demographic characteristics of participants of 2019 survey

|  |  | **Opt-out use of surplus surgical material** | |  |
| --- | --- | --- | --- | --- |
| **Variable** | **Category** | **Yes,**  **N = 400** | **No,**  **N = 540** | ***P* value** |
| Gender (N, %) | Male  Female | 200 (44.1)  200 (41.2) | 254 (55.9)  286 (58.8) | 0.20 |
| Age,  Mean (SD) |  | 45.8 (15.6) | 46.2 (15.6) | 0.67 |
| Marital status (N, %) | Single  Married  Divorced  Widowed | 81 (45.3)  242 (43.2)  45 (37.8)  32 (39.0) | 98 (54.7)  318 (56.8)  74 (62.2)  50 (61.0) | 0.50 |
| Education (N, %) | Primary  Secondary/ professional  Higher | 34 (32.1)  233 (40.2)  133 (52.4) | 72 (67.9)  347 (59.8)  121 (47.6) | < 0.01 |
| Average salary per month per person in the family (Euro) | < 210  211 – 300  301 – 400  401 – 590  > 591 | 63 (33.2)  58 (35.2)  78 (43.1)  64 (49.6)  91 (55.2) | 127 (66.8)  107 (64.8)  103 (56.9)  65 (50.4)  74 (44.8) | < 0.01 |
| Having children under the age of 18 (N, %) | Yes  No | 137 (42.0)  262 (42.8) | 189 (58.0)  350 (57.2) | 0.95 |
| Nationality (N, %) | Latvian  Russian  Other | 254 (45.4)  114 (38.4)  32 (38.1) | 305 (54.6)  183 (61.6)  52 (61.9) | 0.10 |
| Residential status (N, %) | Latvian citizen  Latvian  non-citizen | 356 (44.0)  44 (33.8) | 454 (56.0)  86 (66.2) | 0.03 |
| Working status (N, %) | Governmental sector  Private sector  Not working | 82 (44.3)  198 (46.3)  119 (36.5) | 103 (55.7)  230 (53.7)  207 (63.5) | 0.02 |
| Place of residence (N, %) | Capital city  Another city  Rural area | 155 (49.7)  117 (33.5)  128 (45.9) | 157 (50.3)  232 (66.5)  151 (54.1) | < 0.01 |
