# Supplementary material for: Public awareness of and attitudes towards research biobanks in Latvia
Source: BMC Med Ethics. 2020 Jul 31;21:65. doi: 10.1186/s12910-020-00506-1 (PMC7393882; doi:10.1186/s12910-020-00506-1)
Supplement: Supplementary file 6 — Additional file 6: Supplement Table 6. Relationships between attitude towards sharing of biobank samples among EU states and socio-demographic characteristics of participants of 2019 survey. [file 12910_2020_506_MOESM6_ESM.docx]

Supplement Table 6. Relationships between attitude towards sharing of biobank samples among EU states and socio-demographic characteristics of participants of 2019 survey

|  |  | **Sharing of biobank samples among EU states** | |  |
| --- | --- | --- | --- | --- |
| **Variable** | **Category** | **Yes,**  **N = 607** | **No,**  **N = 218** | ***P* value** |
| Gender (N, %) | Male  Female | 296 (73.1)  311 (74.0) | 109 (26.9)  109 (26.0) | 0.39 |
| Age,  Mean (SD) |  | 45.1 (15.3) | 46.9 (16.4) | 0.13 |
| Marital status (N, %) | Single  Married  Divorced  Widowed | 123 (73.2)  369 (74.7)  77 (75.5)  38 (62.3) | 45 (26.8)  125 (25.3)  25 (24.5)  23 (37.7) | 0.02 |
| Education (N, %) | Primary  Secondary/ professional  Higher | 55 (65.5)  369 (73.5)  182 (76.5) | 29 (34.5)  133 (26.5)  56 (23.5) | 0.15 |
| Average salary per month per person in the family (Euro) | < 210  211 – 300  301 – 400  401 – 590  > 591 | 115 (72.8)  89 (65.4)  109 (73.2)  84 (71.8)  137 (84.0) | 43 (27.2)  47 (34.6)  40 (26.8)  33 (28.2)  26 (16.0) | < 0.01 |
| Having children under the age of 18 (N, %) | Yes  No | 204 (71.8)  401 (74.6) | 80 (28.2)  137 (25.4) | 0.52 |
| Nationality (N, %) | Latvian  Russian  Other | 372 (75.6)  182 (69.7)  52 (73.2) | 120 (24.4)  79 (30.2)  19 (26.8) | 0.22 |
| Residential status (N, %) | Latvian citizen  Latvian  non-citizen | 534 (74.8)  72 (66.1) | 180 (25.2)  37 (33.9) | 0.04 |
| Working status (N, %) | Governmental sector  Private sector  Not working | 123 (74.5)  288 (75.4)  196 (70.5) | 42 (25.5)  94 (24.6)  82 (29.5) | 0.35 |
| Place of residence (N, %) | Capital city  Another city  Rural area | 215 (74.1)  193 (67.2)  198 (80.2) | 75 (25.9)  94 (32.8)  49 (19.8) | < 0.01 |
