# Supplementary material for: Public awareness of and attitudes towards research biobanks in Latvia
Source: BMC Med Ethics. 2020 Jul 31;21:65. doi: 10.1186/s12910-020-00506-1 (PMC7393882; doi:10.1186/s12910-020-00506-1)
Supplement: Supplementary file 7 — Additional file 7: Supplement Table 7. Association of willingness to participate in biobank with awareness, trust and socio-demographic factors – results of logistic regression model. [file 12910_2020_506_MOESM7_ESM.docx]

Supplement Table 7. Association of willingness to participate in biobank with awareness, trust and socio-demographic factors – results of logistic regression model

| Variable | OR | 95% CI | *p* value |
| --- | --- | --- | --- |
| Passive awareness | 0.52 | 0.28; 0.96 | 0.04 |
| Absence of trust | 0.21 | 0.10; 0.44 | < 0.01 |
| Age | 0.97 | 0.95; 0.99 | 0.01 |
| Male gender | 0.90 | 0.50; 1.61 | 0.72 |
| Primary education | 1.31 | 0.31; 5.47 | 0.72 |
| Secondary/ professional education | 1.58 | 0.87; 2.87 | 0.14 |

OR: odds ratio 95% CI: 95% confidence interval
