# Supplementary material for: Public awareness of and attitudes towards research biobanks in Latvia
Source: BMC Med Ethics. 2020 Jul 31;21:65. doi: 10.1186/s12910-020-00506-1 (PMC7393882; doi:10.1186/s12910-020-00506-1)
Supplement: Supplementary file 8 — Additional file 8: Supplement Table 8. Association of preferred type of informed consent (broad over narrow) with awareness, trust and socio-demographic factors – results of logistic regression model. [file 12910_2020_506_MOESM8_ESM.docx]

Supplement Table 8. Association of preferred type of informed consent (broad over narrow) with awareness, trust and socio-demographic factors – results of logistic regression model

| Variable | OR | 95% CI | *p* value |
| --- | --- | --- | --- |
| Passive awareness | 0.92 | 0.52; 1.60 | 0.76 |
| Absence of trust | 0.54 | 0.27; 1.08 | 0.08 |
| Age | 0.99 | 0.97; 1.01 | 0.34 |
| Male gender | 0.76 | 0.44; 1.30 | 0.31 |
| Primary education | 1.97 | 0.54; 7.20 | 0.31 |
| Secondary/ professional education | 0.78 | 0.45; 1.37 | 0.40 |

OR: odds ratio 95% CI: 95% confidence interval
